# Perspectives on non-genetic optoelectronic modulation biointerfaces for advancing healthcare

**DOI:** 10.1007/s44258-024-00030-6

**Published:** 2024-10-21

**Authors:** Aman Majmudar, Saehyun Kim, Pengju Li, Bozhi Tian

**Affiliations:** 1 The James Franck Institute, The University of Chicago, Chicago, IL 60637, USA; 2 Department of Chemistry, The University of Chicago, Chicago, IL 60637, USA; 3 Pritzker School of Molecular Engineering, The University of Chicago, Chicago, IL 60637, USA; 4 The Institute for Biophysical Dynamics, The University of Chicago, Chicago, IL 60637, USA

**Keywords:** Bioelectronics, Photostimulation, Personalized healthcare, Neuromodulation, Cardiac pacemaker

## Abstract

Advancements in optoelectronic biointerfaces have revolutionized healthcare by enabling targeted stimulation and monitoring of cells, tissues, and organs. Photostimulation, a key application, offers precise control over biological processes, surpassing traditional modulation methods with increased spatial resolution and reduced invasiveness. This perspective highlights three approaches in non-genetic optoelectronic photostimulation: nanostructured phototransducers for cellular stimulation, micropatterned photoelectrode arrays for tissue stimulation, and thin-film flexible photoelectrodes for multiscale stimulation. Nanostructured phototransducers provide localized stimulation at the cellular or subcellular level, facilitating cellular therapy and regenerative medicine. Micropatterned photoelectrode arrays offer precise tissue stimulation, critical for targeted therapeutic interventions. Thin-film flexible photoelectrodes combine flexibility and biocompatibility for scalable medical applications. Beyond neuromodulation, optoelectronic biointerfaces hold promise in cardiology, oncology, wound healing, and endocrine and respiratory therapies. Future directions include integrating these devices with advanced imaging and feedback systems, developing wireless and biocompatible devices for long-term use, and creating multifunctional devices that combine photostimulation with other therapies. The integration of light and electronics through these biointerfaces paves the way for innovative, less invasive, and more accurate medical treatments, promising a transformative impact on patient care across various medical fields.

## Introduction

Optoelectronics is a branch of technology that focuses on the interaction between light and electronic devices, encompassing technologies such as light-emitting diodes, solar cells and photo-capacitors. Optoelectronic biointerfaces, formed by integration of optoelectronics with biological tissues, have significantly transformed healthcare [1–10]. One of the most promising applications of optoelectronic biointerfaces lies in nongenetic photostimulation [11, 12]. During photostimulation, optoelectronic biointerfaces convert light into physical stimuli that influence biological activities, ranging from cellular processes to complex physiological functions. By offering precise control over biological processes, photostimulation presents significant advantages over traditional methods such as electrode stimulation, including reduced invasiveness, improved spatial resolution, and the potential for real-time regulation of biological functions. Nongenetic optoelectronic photostimulation contrasts with optogenetics, which employs genetically modified cells with light-sensitive channels for direct modulation using light [13]. These devices offer non-genetic methods for tissue modulation, thereby overcoming the complexity, cost, and ethical concerns associated with optogenetics.

This perspective first provides a focused overview of the latest developments in non-genetic optoelectronic photostimulation, including three main categories: nanostructured phototransducers for cellular stimulation, micropatterned photoelectrode arrays for tissue stimulation, and thin-film flexible photoelectrodes for multiscale stimulation. Each category represents a distinct material and design form factor to harnessing the power of light for medical applications, showcasing the diverse capabilities and potential of optoelectronic technologies. Nanostructured phototransducers enable highly localized stimulation at the cellular level, offering new possibilities for cellular therapy and regenerative medicine. Micropatterned photoelectrode arrays facilitate precise tissue stimulation, which is critical for targeted therapeutic interventions. Thin-film flexible photoelectrodes provide scalable solutions for multiscale stimulation, combining the advantages of flexibility and biocompatibility for a range of medical applications.

While much of the existing literature focuses on the application of optoelectronic devices for neuromodulation [14–17], this perspective will explore future directions and potential applications beyond this area. Optoelectronic biointerfaces hold promise in various medical fields, including cardiology [12], oncology [18–20], wound healing [21], and endocrine [22–24] and respiratory therapies [25]. For instance, optoelectronic devices could revolutionize cardiac therapy by offering minimally invasive solutions for arrhythmia correction and cardiac modulation. In oncology, these devices can enhance the precision of photodynamic therapy, minimizing damage to surrounding tissues and improving patient outcomes [12]. Moreover, optoelectronic biointerfaces have the potential to significantly facilitate wound healing by providing targeted light-based therapies that promote tissue regeneration and reduce healing times. In endocrine and respiratory therapies [25], optoelectronic devices could offer novel approaches for regulating hormonal levels and improving respiratory function through precise light-based interventions.

The integration of optoelectronics in healthcare represents a paradigm shift in medical technology, offering unprecedented opportunities for advancing diagnostics and therapeutics. This perspective aims to highlight the state-of-the-art developments in this rapidly evolving field and to envision the future landscape of optoelectronic biointerfaces in medicine.

## Main

**Fig. 1:**
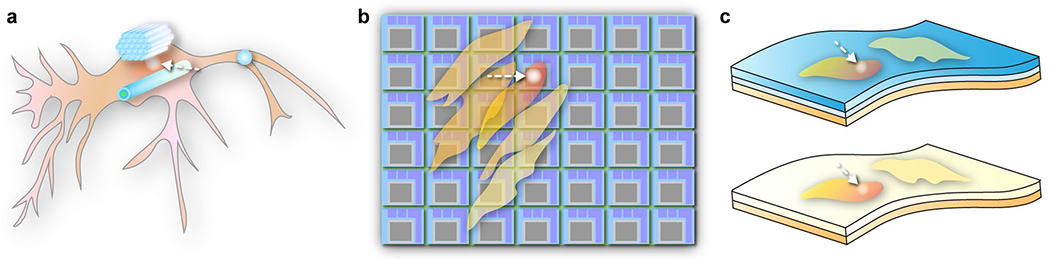
Three types of optoelectronic modulation biointerfaces: **a** nanostructured phototransducers, **b** micropatterned arrays, and **c** thin-film flexible photoelectrodes. The arrows highlight the locations for photostimulation

We will concentrate on three of the most significant and promising forms of photostimulation: nanostructured phototransducers for cellular stimulation, micropatterned photoelectrode arrays for tissue stimulation, and thin-film flexible photoelectrodes for multiscale stimulation (Fig. 1). Photoelectrodes are electrodes that generate an electrochemical potential upon absorbing light. Phototransducers refer to a class of materials that can convert light into various physical stimuli, such as heat or ultrasound and electrical currents.

### Nanostructured phototransducers for cellular stimulation

Nanostructured phototransducers have the capability to convert light into localized heat or electrical currents, enabling precise targeting of individual cells. Conversion of light into heat, called photothermal effect, occurs by non-radiative relaxation of excited electrons that increases the vibrational energy of the lattice. Conversion of light to electrical current, called photoelectric effect, occurs by separation of electron–hole pair and their collection or injection to produce current [7]. Early research into nano-sized semiconductors for biomodulation utilized meso-structured silicon [26] and nanowires to study single-cell electrophysiology. A notable study demonstrated the use of coaxial silicon nanowires for the photoelectrochemical modulation of neuronal activity [27]. These nanowires produce localized electrical currents upon light stimulation, which modulate neuron excitability. The unique structure and properties of these nanowires allow for precise, non-invasive neuromodulation, offering a potential alternative to genetic modification techniques such as optogenetics. These early investigations demonstrated the generation of action potentials and calcium influx in response to high temporal-resolution optical inputs. Additionally, the small size of silicon nanostructures permits their internalization by cells [28], allowing for localized stimulation and modulation of intra- and intercellular networks.

**Fig. 2:**
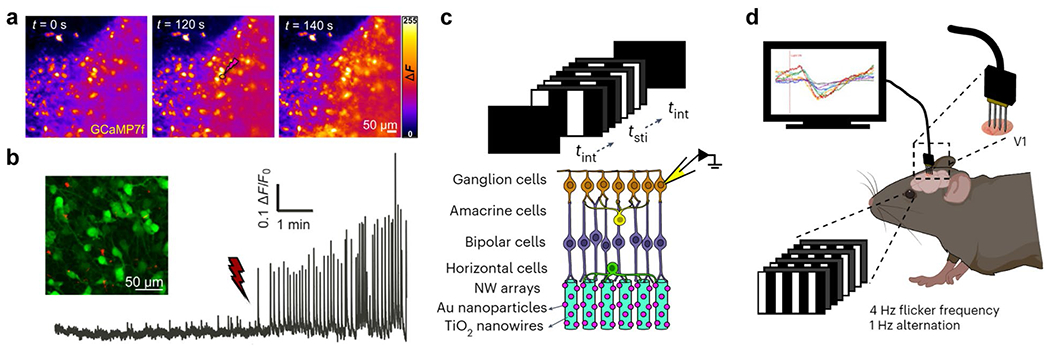
Nanowire-based transducers for optoelectronic modulation interfaces. **a-b** Si nanowires for neuromodulation of the spinal cord dorsal horn. **a** Near-infrared (NIR) light stimulation of nanowires on dorsal OSC neurons enhances network activity. GCaMP7f recording in the spinal cord dorsal horn (DH) with fluorescence levels in false colors at: t = 0, baseline fluorescence (maximal projection of first 100 frames); t = 120, during stimulation (laser spot highlighted with red circle); and t = 140, maximal projection of 100 frames post-stimulation. **b** GluA2 antibody functionalization targets nanowires to DH excitatory pathways. Inset: Confocal image of OSCs expressing neuronal GCaMP7f (green) incubated with GluA2 functionalized nanowires (red). Example of GCaMP7f fluorescent transients showing a significant increase in network activity upon light stimulation of functionalized nanophototransducers (nPDs). **c-d** Au-coated TiO2 nanowire array implantation to study spatial resolution and receptive fields in blind mice. **c** Schematic of ex vivo patch-clamp recording with grating stimuli. **d** Schematic of visually evoked potentials (VEP) recordings. **a** and **b** adapted from reference [29], with permission from the American Association for the Advancement of Science, 2022. **c** and **d** adapted from reference [16], with permission from Springer Nature, 2023

Recent ex vivo research introduced surface-modified coaxial silicon nanowire for targeted neural interfacing [29]. These modified nanowires are capable of precise optical activation and modulation of neurons within mammalian spinal cord explants (Fig. 2a). Functionalized with antibodies against specific synaptic receptors, such as the GluA2 subunit of AMPA-type glutamate receptors, these modified nanowires enable selective targeting and stimulation of excitatory and inhibitory neurons (Fig. 2b). This enhances sensory circuit processing and postsynaptic signaling via light activation. The use of near-infrared light for stimulation with these nanowires allows for deeper tissue penetration and reduces light scattering, highlighting their potential for non-invasive and targeted treatment of neurological conditions characterized by dysfunctional signaling.

The exploration of nanowire array-based structures for translational photostimulation research has shown promising results. For example, gold nanoparticle-coated titania nanowire arrays [16, 30] were used as artificial photoreceptors in mice and monkeys with induced photoreceptor degeneration. The device offers a spatial resolution of 77.5 μm and a temporal resolution of 3.92 Hz in ex vivo retinas (Fig. 2c). In in vivo blind mice, it enabled the detection of gratings and flashing objects at low light intensities (Fig. 2d), with visual acuity between 0.3–0.4 cycles per degree. For monkeys, the arrays remained stable for up to 54 weeks, facilitating the perception of a light beam in visually guided saccade tasks. Additionally, plastic changes in the primary visual cortex suggest potential for restoring vision in patients with photoreceptor degeneration.

Besides using inorganic nanowires or particles, polymeric nanoparticles have been designed to restore vision in advanced-stage retinitis pigmentosa [31]. These nanoparticles mimic the function of natural photoreceptors by converting light into electrical signals to stimulate retinal neurons. The success of this approach in restoring visual function in animal models demonstrates the versatility and potential of nanoscale phototransducers in treating various sensory impairments.

Improvements in this field could include the development of multifunctional nanotransducers capable of delivering drugs and optoelectronic stimulation simultaneously, thereby enhancing therapeutic outcomes. Additionally, optimizing the functionalization of nanowires or nanoparticles with a broader range of specific biomolecules could enable even more precise targeting and modulation of neural circuits. Incorporating advanced imaging techniques for real-time monitoring of cellular responses would provide deeper insights into the mechanisms of action and improve therapeutic precision.

### Micropatterned photoelectrode arrays for tissue stimulation

Micropatterned photoelectrodes have been used for various tissues, particularly in degenerated retinal layers, to replicate the function of natural photoreceptors. This initial proof-of-concept method evoked electrical activity in the degenerated retinal layer when the generated local photocurrent surpassed the activation threshold. Unlike traditional electrode-based stimulation, which often encounters issues with wiring and leads, optoelectronic or photoelectrochemical stimulation provides a wire-free, remotely controlled approach. This method reduces physical strain on the biological interface due to its simple design. Additionally, the use of light allows for precise adjustments in targeted areas, as well as in the intensity and frequency of stimulation. This versatility ensures superior spatiotemporal control, addressing the limitations and challenges associated with pixelated electrode arrays, such as manufacturing complexity and difficulties in portable wireless applications.

**Fig. 3:**
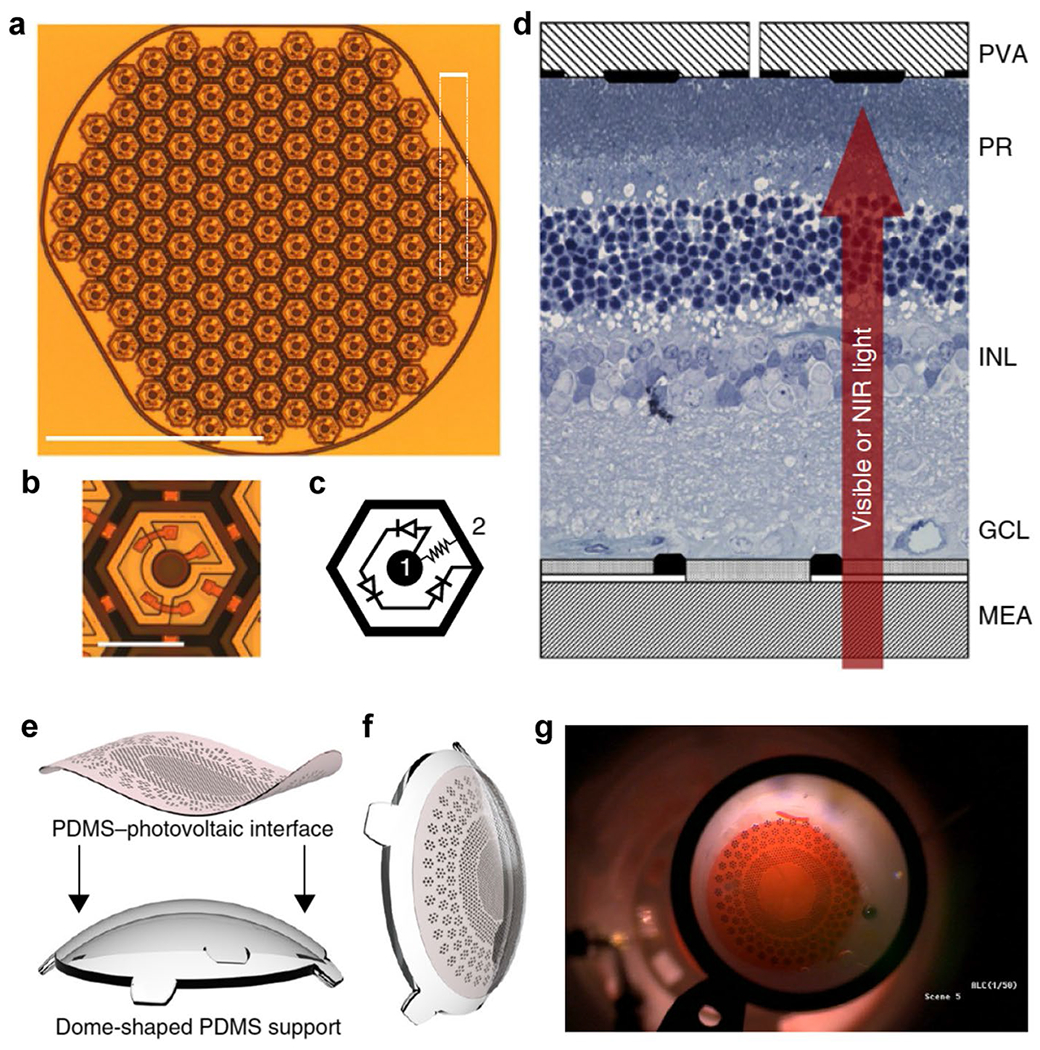
Micropatterned optoelectronic systems for modulation interfaces. **a**-**d** Photovoltaic array and in vitro experimental setup. **a** Image of a single photovoltaic prosthesis module composed of 70-μm-wide pixels separated by 5-μm trenches in a 1-mm-wide hexagonal pattern, with adjacent rows 65 μm apart. Scale bars: top right-hand corner, 65 μm; bottom left-hand corner, 500 μm. **b** Close-up image of a 70-μm-wide pixel. Scale bar, 50 μm. **c** Wiring diagram for each pixel, comprising two to three photodiodes connected in series between the central active (1) and surrounding return (2) electrode. **d** Schematic of a healthy rat retina between a transparent multielectrode array (MEA) recording from the ganglion cell layer (GCL) and the photovoltaic array (PVA). Visible light activates photoreceptors (PR), while much brighter pulsed NIR (880–915 nm) illumination generates biphasic current pulses in the photovoltaic pixels, stimulating cells in the inner nuclear layer (INL). **e**–**g** Foldable and photovoltaic wide-field retinal prosthesis. **e** 3D model of the fabricated PDMS-interface and dome-shaped PDMS support. **f** 3D model of the retinal prosthesis after bonding the PDMS-interface to the PDMS support. **g** Image of POLYRETINA in an epiretinal configuration. **a**-**d** adapted from reference [32], with permission from Springer Nature, 2015. **e**–**g** adapted from reference, with permission from [35] Springer Nature, 2018

The development of photovoltaic retinal prostheses has significantly advanced this technology. Using silicon photodiodes in subretinal arrays (Fig. 3a–d) allows for direct conversion of pulsed near-infrared light into electrical stimulation of retinal neurons, eliminating the need for complex intraocular cables and external power sources [32–34]. This simplifies the surgical procedure and enhances the biocompatibility and longevity of the implants, demonstrating successful in vitro stimulation of both healthy and degenerate retinas and proving the feasibility of high-resolution retinal prostheses. For instance, in one study, subretinal photodiode arrays provided visual acuity close to natural vision by delivering precise and localized stimulation [32]. In another example, high-density photodiode arrays showed the potential for restoring sight by producing electrical signals that closely mimic those generated by natural photoreceptors [34], effectively integrating with the visual pathways of the retina.

In addition to patterned inorganic photoelectrode arrays, organic photoelectrodes have also been widely utilized. Advances include a wide-field spherical array for retinal stimulation, featuring thousands of photovoltaic pixels between a polymeric anode (PEDOT) and a titanium/titanium nitride cathode for high-resolution stimulation of retinal ganglion cells [36]. Another notable example is the foldable and photovoltaic wide-field epiretinal prosthesis, POLYRETINA [35]. This device is designed to cover a large visual field with its hemispherical shape and high pixel density, providing a visual angle of 46.3 degrees and embedding 2215 stimulating pixels (Fig. 3e–g). Its foldable design allows for implantation through a small scleral incision, reducing surgical complexity and improving patient outcomes by enhancing both visual acuity and visual field. POLYRETINA utilizes poly(dimethylsiloxane) (PDMS) as a substrate material, offering transparency, elasticity, and biocompatibility, which ensures long-term functionality and minimizes the risk of cytotoxicity. Successful ex vivo retinal ganglion cell stimulation has validated its potential for restoring vision in patients with retinal dystrophies. Furthermore, this epiretinal stimulation method has demonstrated the ability to enhance and narrow the network-mediated activity of retinal ganglion cells by recruiting the lateral inhibitory network [37]. This approach results in more focal activation of retinal ganglion cells, reducing unwanted activation of axonal pathways and improving the precision of prosthetic vision. By employing non-rectangular voltage pulses, this method effectively recruits inner retinal inhibitory networks, overcoming limitations associated with traditional stimulation techniques.

Developing more sophisticated materials that better mimic the spectral sensitivity of natural photoreceptors will also be crucial. Integrating materials that can absorb a broader spectrum of light, similar to the different types of photoreceptors in the human retina, could enhance the effectiveness of these prostheses [38, 39]. Leveraging advancements in material science to create more durable and biocompatible photoelectrodes will be essential for long-term implantation and functionality. Furthermore, integrating wireless power and data transmission technologies can reduce invasiveness and improve the practicality of these devices for clinical use.

### Thin-film flexible photoelectrodes for multiscale stimulation

Thin-film flexible photoelectrodes, adaptable to tissues such as the brain, retina, sciatic nerve, and heart, offer superior light absorption and electrochemical current injection compared to nanoscale alternatives. These films enable photoelectrochemical stimulation at optical intensities similar to those used in optogenetics. They can be positioned and attached using Van der Waals forces, capillary action, or lock-in mechanisms, which eliminates the need for external bioadhesives. The increased device dimensions help dissipate heat under localized photoexcitation, reducing photothermal effects often seen with nanoparticles. Silicon-based films are particularly notable for their biocompatibility and potential bioresorbability. Enhanced photocurrents and tissue-level stimulation are achieved through engineered p-n or heterojunctions in these membranes. For instance, a gold nanoparticle-decorated p-i-n silicon membrane has facilitated motor cortex stimulation, eliciting limb movement in anesthetized mice [14], while a porosity-based heterojunction membrane has demonstrated control over a rat’s heart rate using light pulses [11]. These techniques hold promise for neuromodulation and the development of new treatments for neurological and cardiac disorders.

**Fig. 4:**
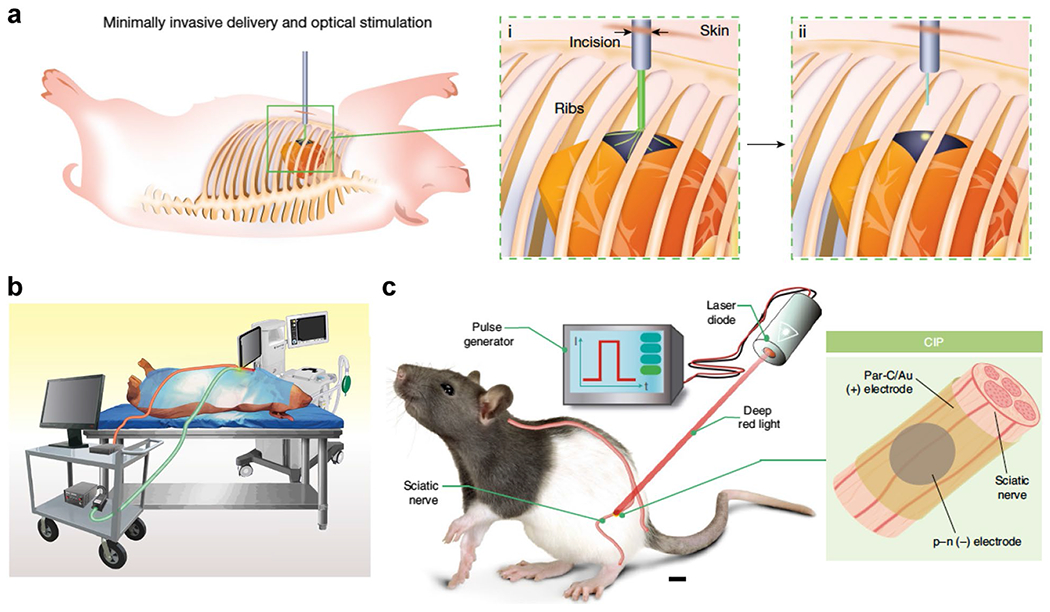
Monolithic optoelectronic systems for modulation interfaces. **a** Minimally invasive approach for closed-thoracic modulation. Schematic representation depicting an incision through the intercostal space, followed by catheter-based delivery of the Si device (i) and subsequent optical-fiber photostimulation (ii). **b** Translational photostimulation on a live pig heart. Schematic illustration of an adult pig in an open-thoracic cardiac-pacing experiment. The setup includes a health-monitoring station, an anesthesia ventilation system, a workstation controlling the laser source, and a recording hub connected to an epicardial flexible MEA. **c** OEPCs wirelessly stimulate the sciatic nerve in vivo. Schematic of the in vivo implanted OEPC photostimulation experiments. Scale bar, 1 cm. Inset details the CIP cuff placement around the nerve, with the primary p–n photoelectrode ( −) and surrounding ( +) return electrode. **a** and **b** adapted from reference [12], with permission from Springer Nature, 2024. **c** adapted from reference [15], with permission from Springer Nature, 2022

Recently, monolithic devices enabling multiscale and random-access stimulation using nanoporous silicon have been developed (Fig. 4a, b) [12]. These devices can precisely modulate individual cardiac cells by adjusting the area of illumination, providing critical insights into electrophysiology under specific spatiotemporal conditions. They have successfully paced rodent heart tissue with low light intensities (0.166 mW/mm^2^ ex vivo and 0.73 mW/mm^2^ in vivo) and demonstrated in vivo overdrive pacing in an adult pig heart using 25 mW/mm^2^ with a 1 ms pulse duration. This technology allows for high-resolution heart modulation at multiple sites, such as the stimulation of 100 distinct positions within a 1 cm^2^ area of a rat heart. Catheter-like devices have been used to deploy and attach silicon-based cardiac stimulators to pig hearts. Once the delivery device is retracted, an optical fiber-coupled endoscope is inserted through the small incision for visually guided optical stimulation. This method demonstrates the feasibility of non-genetic, minimally invasive optical stimulation in a closed-thoracic setting, offering significant potential for heart modulation therapies and transforming the management of cardiac conditions.

When implanting photoelectrode devices for long-term use, it is essential to consider their in vivo stability and light delivery methods. Silicon-based photoelectrodes are suitable for short-term, biodegradable applications, whereas organic semiconductor devices, such as phthalocyanine (H_2_Pc)/PTCDI devices, have shown notable in vivo stability, remaining functional for over 100 days when implanted on rat sciatic nerves, suggesting their potential for chronic neuromodulation (Fig. 4c) [15]. The use of deep red light, penetrating up to 10 mm beneath the skin, facilitates non-invasive activation of these devices, minimizing surgical risks and enhancing trans-tissue stimulation. Photostimulation of an implanted device with 808 nm light, which provides deeper tissue penetration and increased regeneration efficacy, has been shown to effectively promote osteogenesis in cranial defects.

Refining the integration of these thin film devices with advanced imaging and feedback systems would enable real-time monitoring and control. Enhancing the precision and responsiveness of light-based stimulation through adaptive algorithms and machine learning can further optimize therapeutic outcomes. Additionally, exploring the use of new light sources and delivery systems that minimize invasiveness while maximizing effectiveness will be crucial for broad clinical adoption.

## Future directions for optoelectronic modulation devices in biomedical applications

**Fig. 5:**
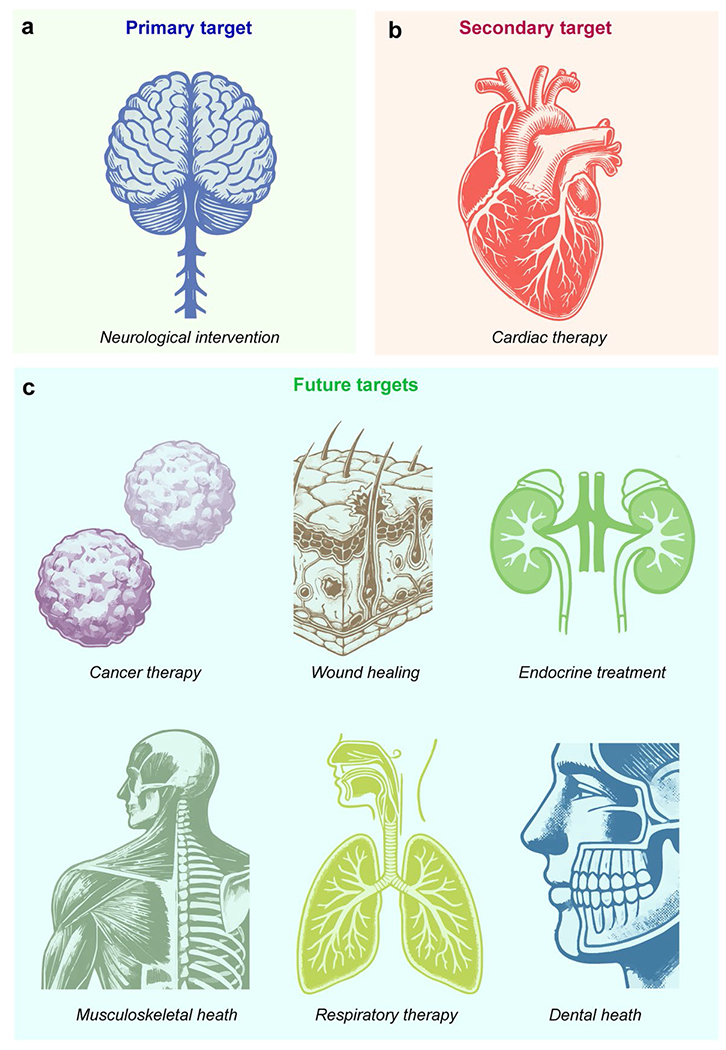
Present and future directions for optoelectronic modulation biointerfaces

Optoelectronic modulation devices are poised to revolutionize biomedical applications by offering precise, non-invasive therapeutic solutions across a range of medical fields (Fig. 5). These devices harness the power of light to modulate biological tissues, presenting opportunities for significant advancements in both diagnosis and treatment. The three types of optoelectronic devices discussed in this article are summarized in Table 1. Future research and development will focus on several key areas to enhance the efficacy and integration of these technologies into clinical practice (Fig. 5c).

### Optoelectronic devices for cardiac therapy

Optoelectronic devices could offer new solutions for cardiac therapy beyond traditional pacemakers. Light-based cardiac therapies, such as optoelectronic devices for photostimulation of cardiac tissue, offer a minimally-invasive approach to modulating heart activity [12]. These devices can deliver precise light pulses to specific areas of the heart to correct arrhythmias or other cardiac dysfunctions. The devices potentially revolutionize the ways to provide temporary heartbeat regulation after open heart surgeries, which are performed more than two-million times worldwide each year. The use of light allows for targeted ablation of arrhythmogenic tissue, reducing the risk of complications associated with traditional electrical ablation techniques, such as damages to valves and heart conduction [40]. The compatibility enables the integration of optoelectronic therapy into robotic-assisted surgeries, allowing treatments to be administered automatically and remotely.

Future research should explore integrating optoelectronic devices with advanced imaging technologies to guide and monitor cardiac therapies more precisely. For instance, coupling these devices with MRI and CT imaging could enhance the accuracy of device placement and the real-time assessment of therapeutic outcomes. Developing wireless and battery-free optoelectronic devices that can be implanted with minimal invasiveness will also be essential. Recent advances in miniaturized, fully implantable devices capable of both electrical and optical stimulation show promise in this area [41], as they can operate without bulky batteries or external power sources, allowing for more naturalistic and long-term studies in small animal models.

Additionally, creating personalized cardiac therapy programs based on continuous data monitoring and AI analysis could improve patient outcomes. These programs could leverage data from implanted optoelectronic devices to adapt therapies in real time, optimizing treatment efficacy and minimizing side effects. Integration of optoelectronic pacing systems with biological pacing may also present a future direction. Biological pacing aims to create pacemakers from the patient’s own cells, offering a more natural rhythm regulation than electronic devices [42]. Combining this approach with optoelectronic technology could lead to hybrid systems that provide both biological and light-based modulation, enhancing therapeutic flexibility and effectiveness.

#### Targeted cancer therapy

Optoelectronic devices hold significant potential in oncology, particularly in targeted cancer treatment (Fig. 5c) [18–20]. Photodynamic therapy (PDT) employs light-sensitive compounds activated by specific wavelengths of light to generate reactive oxygen species (ROS) that selectively kill cancer cells. As optoelectronic devices can induce photoelectrochemical effects in tissues, and ROS can be produced through oxidative or reductive processes, these devices offer the potential to deliver PDT with high precision, minimizing damage to surrounding healthy tissues. To effectively target cancerous cells at the cellular level within a tissue, use of nanostructured phototransducers would be the optimal method. These nanoscale phototransducers may be modified with cancer-specific biomarkers to achieve selective targeting [43]. The integration of optoelectronic sensors enables real-time monitoring of therapy effectiveness, allowing for personalized treatment adjustments.

Future research should focus on enhancing the specificity and efficiency of light-sensitive semiconductors or their coatings to target a broader range of cancer types. Developing multifunctional optoelectronic devices that combine PDT with other treatment modalities, such as chemotherapy or immunotherapy, could improve therapeutic outcomes. Additionally, creating portable and user-friendly devices for outpatient cancer treatment could enhance patient accessibility and comfort.

### Advanced wound healing

Optoelectronic devices have the potential to revolutionize wound care by offering therapeutic capabilities through light modulation (Fig. 5c) [44]. Light-based therapies, such as low-level laser therapy (LLLT) and light-emitting diode (LED) therapy, promote wound healing by increasing collagen production and enhancing blood flow. Incorporating thin-film flexible photoelectrode, matching the length scale of wounds, into bandages or wearable patches may provide additional synergistic effects such as electrotaxis or ROS-triggered tissue regeneration. Additionally, developing sophisticated optoelectronic wound healing devices that integrate multiple therapeutic modalities, including drug delivery and mechanical stimulation, would enhance healing efficacy. Designing wearable devices for home use will improve patient convenience and compliance [45]. Additionally, further research into the mechanisms underlying light-based therapies can lead to more targeted and effective treatment protocols.

### Implantable optoelectronic devices for endocrine disorders

Managing endocrine disorders, such as thyroid or adrenal gland dysfunctions, often requires precise modulation of hormone levels. Implantable optoelectronic devices could provide targeted photostimulation to modulate endocrine function (Fig. 5c) [22–24]. For instance, an optoelectronic device could stimulate hormone production or release in an optogenetic manner in response to light, offering a more controlled approach to managing endocrine disorders. While it remains uncertain if non-genetic photoelectrochemical stimulation could directly achieve this, it represents a promising direction for future research.

Enhancing the accuracy and longevity of implantable optoelectronic devices for endocrine modulation is critical. Developing biocompatible and minimally invasive devices that can be easily implanted and maintained will be essential for practical applications. Additionally, integrating these devices with automated drug delivery systems could create a closed-loop system for optimal endocrine disorder management. Future efforts should focus on refining the materials and designs of these devices to improve their performance and reliability over extended periods. Investigating the underlying mechanisms of photostimulation and its effects on endocrine tissues will also be vital in advancing this technology and ensuring its efficacy and safety in clinical settings.

### Optoelectronic devices for musculoskeletal health

The management of musculoskeletal conditions, such as osteoarthritis and muscle injuries, could be enhanced by optoelectronic devices [46–48]. Light-based therapies have shown promise in reducing pain and promoting tissue repair in musculoskeletal disorders. Optoelectronic devices, if integrated as micropatterned photoelectrode arrays, could deliver the therapies in a targeted and controllable manner (Fig. 5c). Photoelectrode arrays could provide site-specific phototherapy or alternatively, provide 2D electronic read-out of musculoskeletal tissue conditions. Possible advancements could include developing wearable optoelectronic devices that integrate seamlessly with the patient’s body, providing continuous and adjustable light therapy for musculoskeletal health. Enhancing the precision and control of light delivery mechanisms can improve therapeutic outcomes. Additionally, researching the underlying mechanisms of light-based therapies can lead to the development of more targeted and effective treatment protocols.

### Optoelectronic devices for respiratory therapy

Optoelectronic technologies offer promising potential for targeted respiratory therapies, such as phototherapy, to reduce airway inflammation and improve lung function (Fig. 5c) [49, 50]. These devices can deliver precise light doses to specific areas of the respiratory tract, providing a non-invasive approach to managing conditions like chronic obstructive pulmonary disease (COPD) and asthma. Given the unique challenges posed by conditions like COVID-19-induced acute respiratory distress syndrome (ARDS), also known as CARDS, there is an urgent need to develop advanced optoelectronic devices [25]. These should be capable of delivering targeted PDT to treat viral-induced damage and inflammation. The injection of semiconductor-based photosensitizers, followed by targeted photoactivation, could serve as a novel therapeutic approach, leveraging the ability of PDT to generate reactive oxygen species and destroy pathogens, including SARS-CoV-2.

Research should focus on enhancing the accuracy and reliability of these devices under various conditions, ensuring they can provide comprehensive respiratory therapeutic capabilities. Additionally, integrating these devices with telemedicine platforms can enable remote monitoring and management of respiratory conditions, improving patient outcomes and accessibility to care. This integration is crucial for enhancing the treatment of severe respiratory conditions, especially in resource-limited settings.

### Optoelectronic devices for dental health

Dental health is another area where optoelectronic devices could make a significant impact (Fig. 5c). Light-based therapies could be used to treat cavities, remove diseased tissue, and promote the healing of oral wounds with greater precision and less discomfort compared to traditional methods [51, 52]. Optoelectronic devices could be designed to deliver these therapies in a targeted and controlled manner. Future research should focus on developing more advanced optoelectronic devices for dental health that provide precise and effective light-based treatments. Creating compact and user-friendly devices that can be easily used by dental professionals will improve adoption and patient outcomes. Additionally, integrating these devices with digital dental health platforms can provide a more comprehensive and personalized approach to dental care.

### Optoelectronic devices for trainable biointerface

Biological training processes such as muscle strengthening and synaptic enhancement have inspired the creation of trainable bioelectronics. This trainability enables bioelectronics to adapt to environmental changes on-demand, improving their functionality and lifespan [53, 54]. A novel approach to building trainable biointerfaces involves incorporating living materials at the tissue-electronics junction, leading to the concept of ‘living bioelectronics’. The living materials, composed of bacteria encapsulated within a hydrogel matrix, can imbue the interface with life-like, stimuli-responsive and trainable characteristics [55]. While an existing living bioelectronics utilized planer electrodes to electrochemically control the bioactivity of the living interface [56], we propose that optoelectronics could also be a powerful tool for training and modulation of living materials. For instance, by embedding nanostructured phototransducers with live bacteria in the hydrogel, it may be possible to influence bacterial metabolism through light exposure, reconfiguring the properties of the biointerface [57]. Exploring how these phototransducers interact with the bacteria within the matrix, and correlating bacterial responses with desired biointerface properties, could pave the way for integration of optoelectronics to create light-trainable living biointerfaces.

## Summary

Our perspective discusses the transformative role of non-genetic optoelectronic modulation biointerfaces in modern medicine. These biointerfaces seamlessly blend light and electronics to offer potentially groundbreaking solutions for precise stimulation and monitoring of biological systems, significantly advancing both diagnostics and therapeutics.

We categorize the advancements into three pivotal areas: nanostructured phototransducers for cellular stimulation, micropatterned photoelectrode arrays for tissue stimulation, and thin-film flexible photoelectrodes for multiscale stimulation. Nanostructured phototransducers allow for highly localized stimulation, which is vital for cellular therapies and regenerative medicine. Micropatterned photoelectrode arrays enable precise tissue-level stimulation, essential for targeted therapeutic interventions. Meanwhile, thin-film flexible photoelectrodes provide scalable solutions adaptable to various tissues, ensuring a wide range of medical applications.

Moreover, our perspective envisions these technologies extending far beyond neuromodulation. We foresee their potential in cardiology, oncology, wound healing, and endocrine and respiratory therapies. Future advancements will likely focus on integrating optoelectronic devices with sophisticated imaging and feedback systems for precise monitoring and control. Additionally, the development of wireless and biocompatible devices for long-term use, along with multifunctional devices that combine photostimulation with other therapeutic modalities, represents an exciting frontier in medical technology.

Nonetheless, several limitations of optoelectronic biointerfaces must be addressed for practical biomedical applications. Achieving minimal invasiveness remains a significant hurdle, as many optoelectronic devices need to be integrated into or near sensitive tissues, which can lead to complications or discomfort. Additionally, the degradation of silicon electronics over time poses a challenge for long-term use, as silicon components can suffer from wear and reduced functionality when exposed to biological environments. Addressing light penetration issues is also critical, as tissues often limit the depth and effectiveness of optical stimulation or imaging. Future advancements must focus on developing more biocompatible materials [58], enhancing the durability of electronic components [59], and improving techniques for effective light delivery and signal detection through tissue. Upon addressing these concerns, optoelectronic biointerfaces promise to revolutionize patient care by making treatments more effective, less invasive, and personalized, setting a new standard in medical care.

## Figures and Tables

**Table 1:** Comparative table of optoelectronic devices

Device Type	Device Materials	Scale	Operating Principle	Applications	Ref
**Nanostructured Phototransducers**	Coaxial silicon nanowires	~ 200 nm (diameter) ~ 5 μm (length)	Coaxial silicon nanowires produce localized electrical currents upon light stimulation, modulating neuron excitability	Neuromodulation at the single neurons and spinal explants	[27, 29]
	Gold nanoparticle-coated titania nanowire arrays	~ 200 nm (diameter) ~ 2 μm (length)	Nanowire arrays generate capacitive and faradaic photocurrents, providing artificial photoreceptor functions	Neuromodulation, vision restoration in degenerated retinas	[16, 30]
	Polymeric nanoparticles	~ 182 nm (diameter)	The nanoparticles mimic natural photoreceptors by converting light into electrical signals to stimulate retinal neurons	Vision restoration	[31]
**Micropatterned Photoelectrode Arrays**	Silicon photodiodes	~ 50 μm – 140 μm	Photovoltaic retinal prosthesis arrays convert near-infrared light into electrical stimulation of retinal neurons	Retinal prosthesis for high visual acuity restoration in patients with retinal degeneration	[32, 33]
	P_3_HT:PCBM	~ 80 μm	Thousands of photovoltaic pixels provide high-resolution stimulation of retinal ganglion cells	High-resolution retinal stimulation for vision restoration in retinal ganglion cells	[36]
	P_3_HT:PCBM	~ 100 μm	Foldable wide-field photovoltaic pixels stimulate retinal ganglion cells, mimicking the function of natural photoreceptors	Vision restoration in patients with retinal dystrophies through broad visual field stimulation	[35]
**Thin-Film Flexible Photoelectrodes**	Porosity-based silicon membranes	~ 2 μm (thickness) ~ 4 mm (diameter)	Porosity-based heterojunctions generate and separate photocarriers for capacitive current injection	Ex vivo rat heart pacing, sciatic nerve stimulation	[11]
	Porosity-based silicon membranes	~ 5 μm (thickness) ~ 1 – 20 mm (diameter)	Photocarriers were separated and generated at heterojunctions and space charge layers for capacitive stimulation	Multiscale cardiac stimulation on single cells, monolayers, ex vivo cardiac tissues and in vivo rodents’ and pigs’ heart	[12]

## Data Availability

Not applicable.

## Highlights

- Non-genetic optoelectronic biointerfaces revolutionize healthcare by enabling precise biological stimulation and monitoring.
- Key methods include nanostructured phototransducers, micropatterned arrays, and thin-film flexible electrodes.
- Future advancements will integrate imaging, develop wireless devices, and create multifunctional therapies, transforming patient care.
